# Supplementary material for: Electrochemical topological transformation of polysiloxanes
Source: Commun Chem. 2021 Sep 15;4:130. doi: 10.1038/s42004-021-00570-7 (PMC9814237; doi:10.1038/s42004-021-00570-7)
Supplement: Supplementary file 1 — Description of Additional Supplementary Files [file 42004_2021_570_MOESM1_ESM.pdf]

Description of Additional Supplementary Files

File Name: Supplementary Movie 1

Description: An oily liquid mixture of $\mathbf{S_L}$ and [EMI][TFSI] (weight ratio: $\mathbf{S_L}$/[EMI][TFSI] = 70/30).

File Name: Supplementary Movie 2

Description: Initiation of electrochemical oxidation of $\mathbf{S_L}$ upon applying constant potential at 1.0 V by inserting carbon electrodes to a mixture of $\mathbf{S_L}$ and [EMI][TFSI] (weight ratio: $\mathbf{S_L}$ /[EMI][TFSI] = 70/30).

File Name: Supplementary Movie 3

Description: Increase of viscosity during electrochemical oxidation of $\mathbf{S_L}$ upon applying constant potential at 1.0 V to a mixture of $\mathbf{S_L}$ and [EMI][TFSI] (weight ratio: $\mathbf{S_L}$ /[EMI][TFSI] = 70/30).

File Name: Supplementary Movie 4

Description: Nonflowable material formed on a vial after electrochemical oxidation of $\mathbf{S_L}$ upon applying constant potential at 1.0 V to a mixture of $\mathbf{S_L}$ and [EMI][TFSI] (weight ratio: $\mathbf{S_L}$ /[EMI][TFSI] = 70/30).

File Name: Supplementary Movie 5

Description: Change in the flowability of IN70 on an inverted vial upon photoirradiation.
