# Supplementary material for: Electrochemical topological transformation of polysiloxanes
Source: Commun Chem. 2021 Sep 15;4:130. doi: 10.1038/s42004-021-00570-7 (PMC9814237; doi:10.1038/s42004-021-00570-7)
Supplement: Supplementary file 2 — Supplementary Information [file 42004_2021_570_MOESM2_ESM.pdf]

Supplementary Information

Electrochemical topological transformation of polysiloxanes

Minami Oka and Satoshi Honda[*]

Department of Basic Science, Graduate School of Arts and Sciences, The University of Tokyo, 3-8-1 Komaba, Meguro-ku, Tokyo 153-8902, Japan.

Experimental Section

Materials

Dimethylphenylsilanol (Wako, > 99%), (n-Bu)$_4$NClO$_4$ (TCI, > 99%), D3 (TCI), and other reagents were used as received unless otherwise noted. Bromo-appended lophine (**1**),[1] vinyl-appended lophine (**L**$_V$),[2] hydrosilane-appended lophine (**L**$_S$),[1] linear monodisperse oligo(dimethyl siloxane) with lophine end groups (**M**$_L$) (Number averaged molecular weight (M_n), weight averaged molecular weight (M_w), peak molecular weight (M_p), and dispersity ($Đ = M_w/M_n$) measured by size exclusion chromatography (SEC) calibrated with polystyrene standards; M_n = 1700, M_w = 1700, M_p = 1700, $Đ$ = 1.05)[2] were synthesized according to the previously reported procedures. 1,3,5-Tris(dimethylhydroxysilyl)benzene[3] and 1-(3,5-bis(trifluoromethyl)phenyl)-3-(3-(trifluoromethyl)phenyl)urea (U(3CF$_3$))[4] were synthesized according to modified procedures in the literatures.

Synthesis of linear PDMS with a vinyl end group

Into a flask was prepared a mixture of dimethylphenylsilanol (155 μL, 1.0 mmol) and D3 (2.22 g, 10 mmol) in THF (3 mL). A separately prepared THF solution (0.50 mL) of TBD (14 mg, 0.10 mmol) was added to the mixture and the resulting solution was stirred at 25 °C. After 60 min, pyridine (0.81 mL, 10 mmol) and chlorodimethylvinylsilane (1.36 mL, 10 mmol) was added in this order and the mixture was further stirred for 13 h. The mixture was poured into an excess amount of H_2O/hexane and aqueous layer was separated and extracted with hexane. The combined hexane phases were washed with water, dried over Na_2SO_4, and concentrated to dryness. The crude oily product obtained was then washed with MeOH and dissolved in $CHCl_3$. The resulting solution was

concentrated and dried under reduced pressure. The product, vinyl-end-functionalized PDMS, was isolated in a yield of 1.52 g. ^{1}H NMR (500 MHz, acetone-d_6) δ ppm −0.10–0.16 (–SiO(CH_3)$_2$–), 0.18 (–Si(CH_3)$_2$CH=CH$_2$), 0.40 (ArSi(CH_3)$_2$O–), 5.79 (–Si(CH$_3$)$_2$CH=CH_2), 5.96 (–Si(CH$_3$)$_2$CH=CH_2), 6.17 (–Si(CH$_3$)$_2$CH=CH$_2$), 7.87 (ArH).

Synthesis of linear PDMS with a lophine end group (P$_L$)

Into a flask was prepared a mixture of vinyl-end-functionalized PDMS (1.40 g, 0.467 mmol), **L$_S$** (497 mg, 1.40 mmol), Karstedt's catalyst (0.10 mL) and THF (10 mL), and the resulting solution was refluxed for 18 h. After cooling to room temperature, the mixture was concentrated and the residue was diluted with hexane, filtered, and filtrate was concentrated to dryness. The crude product was purified by dialysis against CHCl$_3$ to afford **P$_L$** as a white solid. The yield was 1.2 g (86%). ^{1}H NMR (500 MHz, acetone-d_6) δ ppm −0.07–0.16 (–SiO(CH_3)$_2$–), 0.31 (–Si(CH_3)$_2$CH$_2$CH$_2$–), 0.40 (ArSi(CH_3)$_2$O–), 0.53 (–Si(CH$_3$)$_2$CH_2CH$_2$–), 0.78 (–Si(CH$_3$)$_2$CH$_2$CH_2–), 7.20–8.15 (ArH), 11.72 (–NH). M_n, M_w, M_p, and $Đ$ measured by SEC calibrated with polystyrene standards; M_n = 3300, M_w = 4500, M_p = 4200, $Đ$ = 1.37.

Synthesis of three-armed star-shaped PDMS with vinyl end groups.

Into a flask was prepared a mixture of U(3CF$_3$) (83.2 mg, 0.20 mmol), NaH (4.8 mg, 0.20 mmol), and THF (0.80 mL). To this mixture, 1,3,5-tris(dimethylhydroxysilyl)benzene (20 mg, 0.20 mmol for OH groups) was added and agitated until homogeneous solution was obtained. A separately prepared THF solution (12 mL) of D3 (10.0 g, 45.0 mmol) was added to the mixture and the resulting solution was stirred at room temperature (25 °C). After 60 min, THF solution (0.50 mL) of benzoic acid (244 mg, 2.0 mmol) was added and the mixture was further stirred for 150 min. After evaporation to dryness, the residue

was washed sequentially with MeCN and acetone. The resulting PDMS was dried under reduced pressure to afford hydroxy-terminated three-armed star-shaped PDMS, which was directly subjected to the following end-capping reaction with chlorodimethylvinylsilane. Thus, a part of the obtained PDMS (1.0 g, 0.133 mmol for OH end groups), THF (5 mL), pyridine (640 μL, 8.0 mmol), and chlorodimethylvinylsilane (360 μL, 2.7 mmol) was added in this order and the mixture was further stirred for 17 h. The mixture was poured into an excess amount of H_2O/hexane. The hexane phase was washed with water, dried over Na_2SO_4, and concentrated to dryness. The obtained crude oily product was washed sequentially with MeOH and acetone and dried under reduced pressure to afford vinyl-terminated three-armed star-shaped PDMS as a colorless oil. The yield was 940 mg. The procedure was repeated twice and totally 1.8 g of three-armed star-shaped PDMS with vinyl end groups was synthesized. ^{1}H NMR (500 MHz, $CDCl_3$) δ ppm −0.20–0.90 (–SiO(CH_3)$_2$–, –Si(CH_3)$_2$CH=CH$_2$, ArSi(CH_3)$_2$O–), 5.73 (–Si(CH$_3$)$_2$CH=CH_2), 5.93 (–Si(CH$_3$)$_2$CH=CH_2), 6.12 (–Si(CH$_3$)$_2$CH=CH$_2$), 7.76 (ArH). M_n, M_w, M_p, and $Đ$ measured by SEC calibrated with polystyrene standards; $M_n = 22300$, $M_w = 26600$, $M_p = 21500$, $Đ = 1.20$.

Synthesis of three-armed star-shaped PDMS with lophine end groups (S_L).

In a typical procedure, a mixture of the vinyl-terminated three-armed star-shaped PDMS (1.7 g, 0.228 mmol for vinyl groups), $\mathbf{L_S}$ (403 mg, 1.14 mmol), Karstedt's catalyst (0.10 mL) and THF (5 mL) was prepared in a flask and refluxed for 20.5 h. After cooling to room temperature, the mixture was concentrated, washed with MeOH and acetone. The resulting oil product containing small droplets of MeOH was then dissolved in $CHCl_3$ and the resulting homogeneous mixture was concentrated to dryness under reduced pressure

to afford $\mathbf{S_L}$ as a white solid. The yield was 1.45 g (83%). ^{1}H NMR (500 MHz, $CDCl_3$) δ ppm −0.10–0.40 (–SiO(CH_3)$_2$–, –Si(CH_3)$_2CH_2CH_2$–, ArSi(CH_3)$_2$O–), 0.44 (–Si(CH_3)$_2$CH_2CH$_2$–), 0.68 (–Si(CH_3)$_2$CH$_2$CH_2–), 7.30–7.92 (Ar*H*), 9.25 (–N*H*). M_n, M_w, M_p, and $Đ$ measured by SEC calibrated with polystyrene standards; M_n = 22000, M_w = 25300, M_p = 21400, $Đ$ = 1.17.

NMR Measurements

^{1}H NMR spectra were recorded on a Bruker AVANCE III spectrometer operating at 500 MHz. $CDCl_3$ or DMSO-d_6 was used as the solvent and chemical shifts were reported relative to tetramethylsilane (TMS) (δ = 0.00 ppm) or solvent residual signals.

ESR measurements

ESR spectra were recorded on a JEOL JES-TE3000 spectrometer at room temperature. Photoirradiation was performed using a consumer use blue laser pointer (λ = 400–410 nm) from a window equipped with a sample insertion opening.

Mass Spectrometric Analyses

Mass spectra (APCI) was recorded using an Advion Expression CMS-L mass spectrometer. Electrospray ionization (ESI) high-resolution mass (HRMS) spectra were recorded at Advanced Instrumental Analysis Unit in Graduate School of Agricultural and Life Sciences at the University of Tokyo.

UV-vis absorbance measurements

UV-vis absorption spectra were recorded on a SHIMADZU UV-1900i spectrometer. Time-course measurement was performed by plotting absorbance at the wavelength of

568 nm against time. For time-dependent UV-vis measurements upon applying potential, two mechanical pencil leads were put through a cap with silicone rubber for a quartz cell, and connected a lead wire to each of them as shown in Supplementary Figure 6.

SEC Measurements

SEC measurements were performed using a Waters e-2695 high-speed liquid chromatograph equipped with RI and UV detectors. THF was used as the eluent and a Shodex KF-603 column (flow rate: 0.50 mL/min) or two series-connected TSKgel SuperMultipore HZ-H columns (flow rate: 0.35 mL/min) were employed at 40 °C. The calibration curve was obtained with TSK standard polystyrenes (Tosoh Co.); the M_w(LS)s were 189000, 37200, 9490, 2500, 589, respectively.

Supplementary Scheme 1. Synthesis of model compound. Synthesis of $\mathbf{M_L}$ by hydrosilylation of 1,1,3,3,5,5,7,7,9,9,11,11,13,13-tetradecamethylheptasiloxane with $\mathbf{L_V}$.

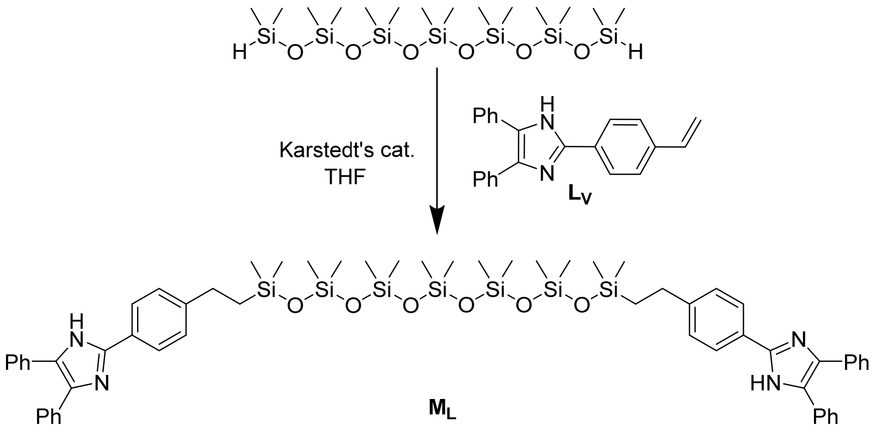

Supplementary Scheme 2. Synthesis of $\mathbf{P_L}$. a Synthesis of vinyl-end-functionalized PDMS by ROP of D3 initiated from dimethylphenylsilanol. **b** Synthesis of $\mathbf{P_L}$ by hydrosilylation of vinyl-end-functionalized PDMS with $\mathbf{L_S}$.

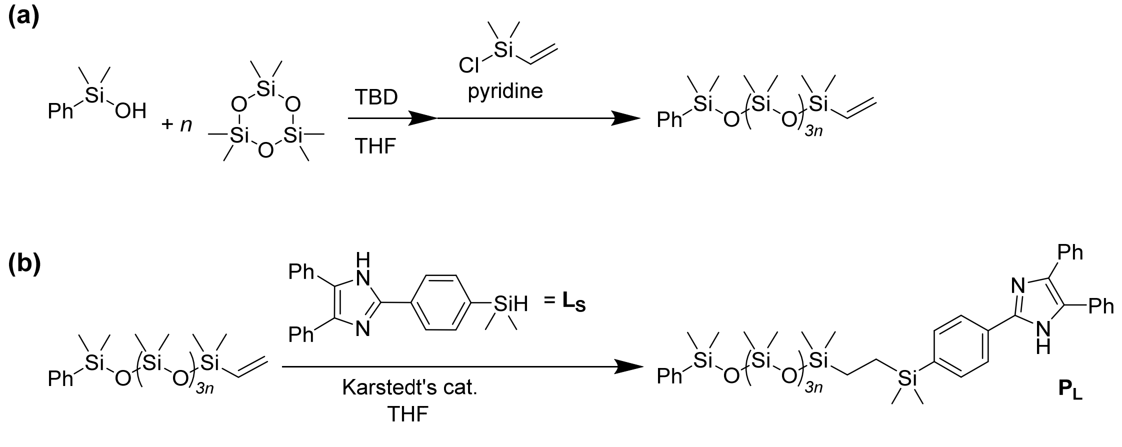

Supplementary Scheme 3. Synthesis of network PDMS linked with HABIs in the chains (N). a Synthesis of three-armed star-shaped PDMS with vinyl end groups by ROP of D3 initiated from trifunctional silanol terminated with chlorodimethylvinylsilane and following hydrosilylation with $\mathbf{L_S}$ to synthesize $\mathbf{S_L}$. **b** Synthesis of **N** by electrochemical oxidation of $\mathbf{S_L}$.

(a)

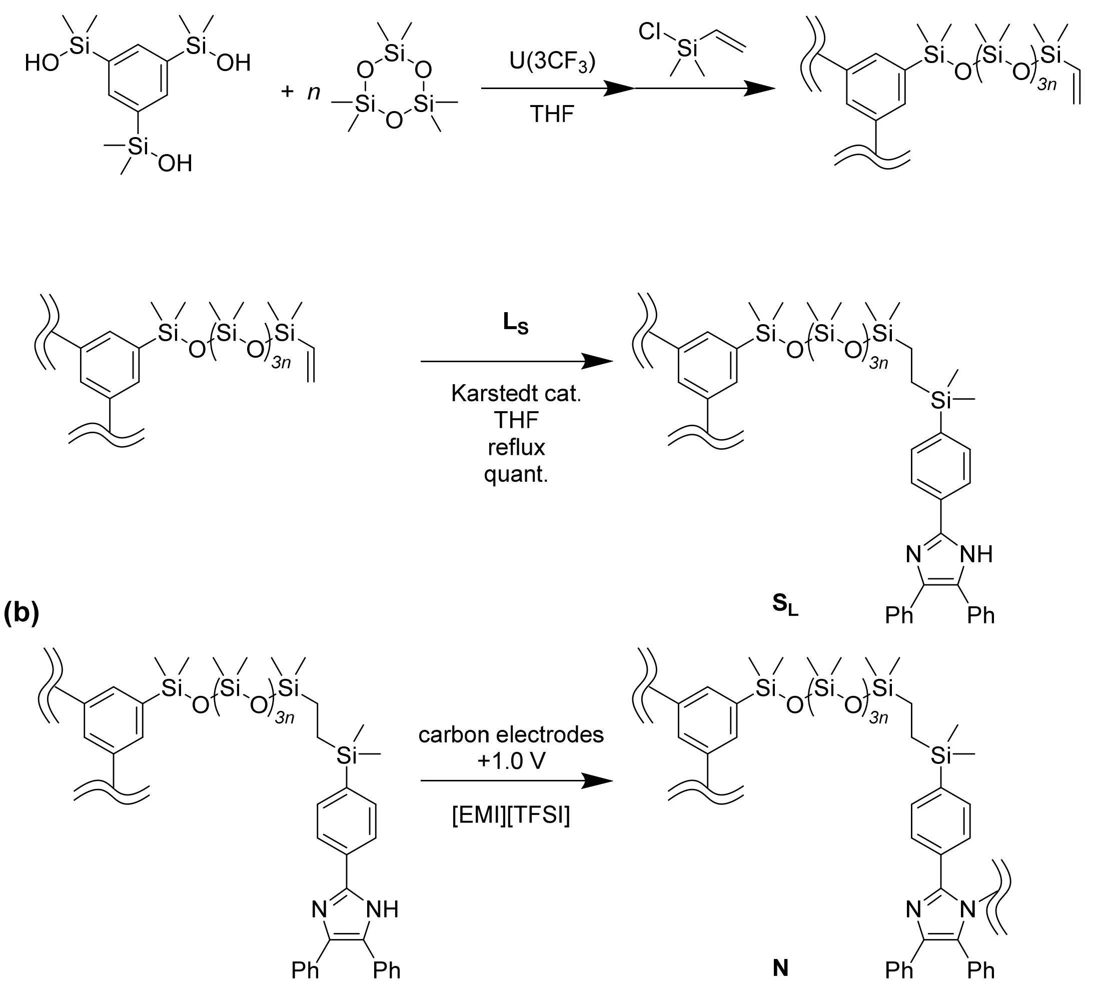

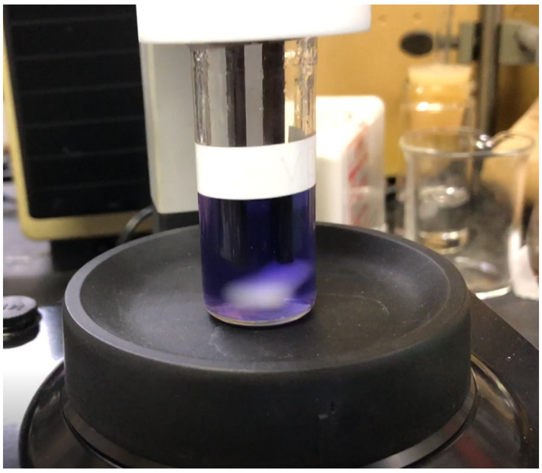

Supplementary Figure 1. Electrochemical oxidation of 1. Photograph of a reaction mixture of **1** upon applying a potential of 1.3 V.

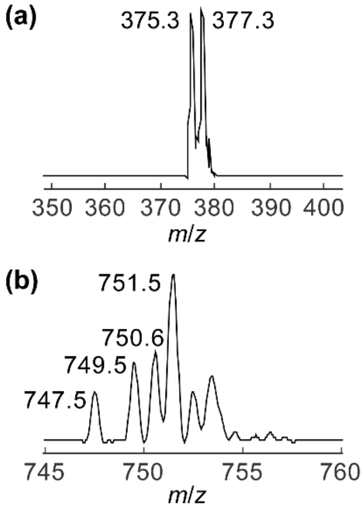

Supplementary Figure 2. Characterization of dimerization based on mass spectrometry. APCI mass spectra of (a) **1** and (b) **2**.

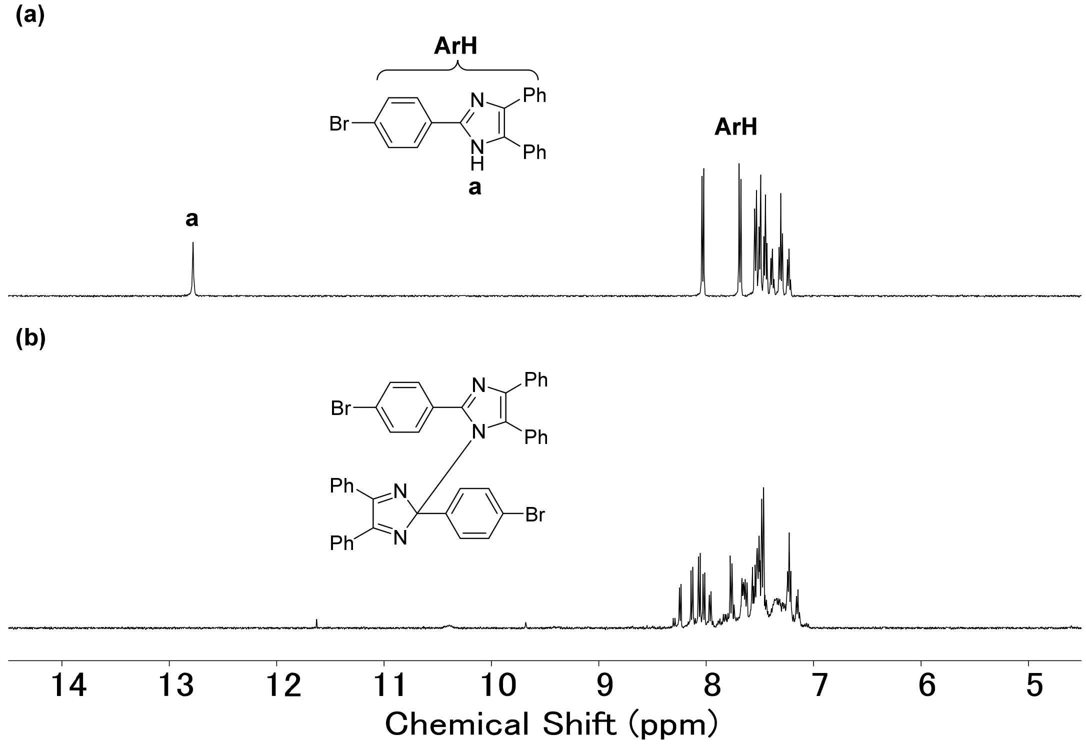

Supplementary Figure 3. Characterization of dimerization based on NMR spectrometry. 500 MHz ^{1}H NMR spectra of (a) **1** and (b) **2** (DMSO-d_6).

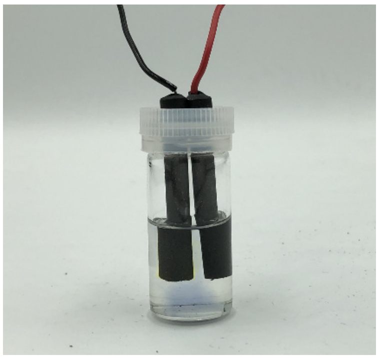

Supplementary Figure 4. Electrochemical oxidation of $\mathbf{M_L}$. Photograph of reaction mixture of $\mathbf{M_L}$ upon applying potential.

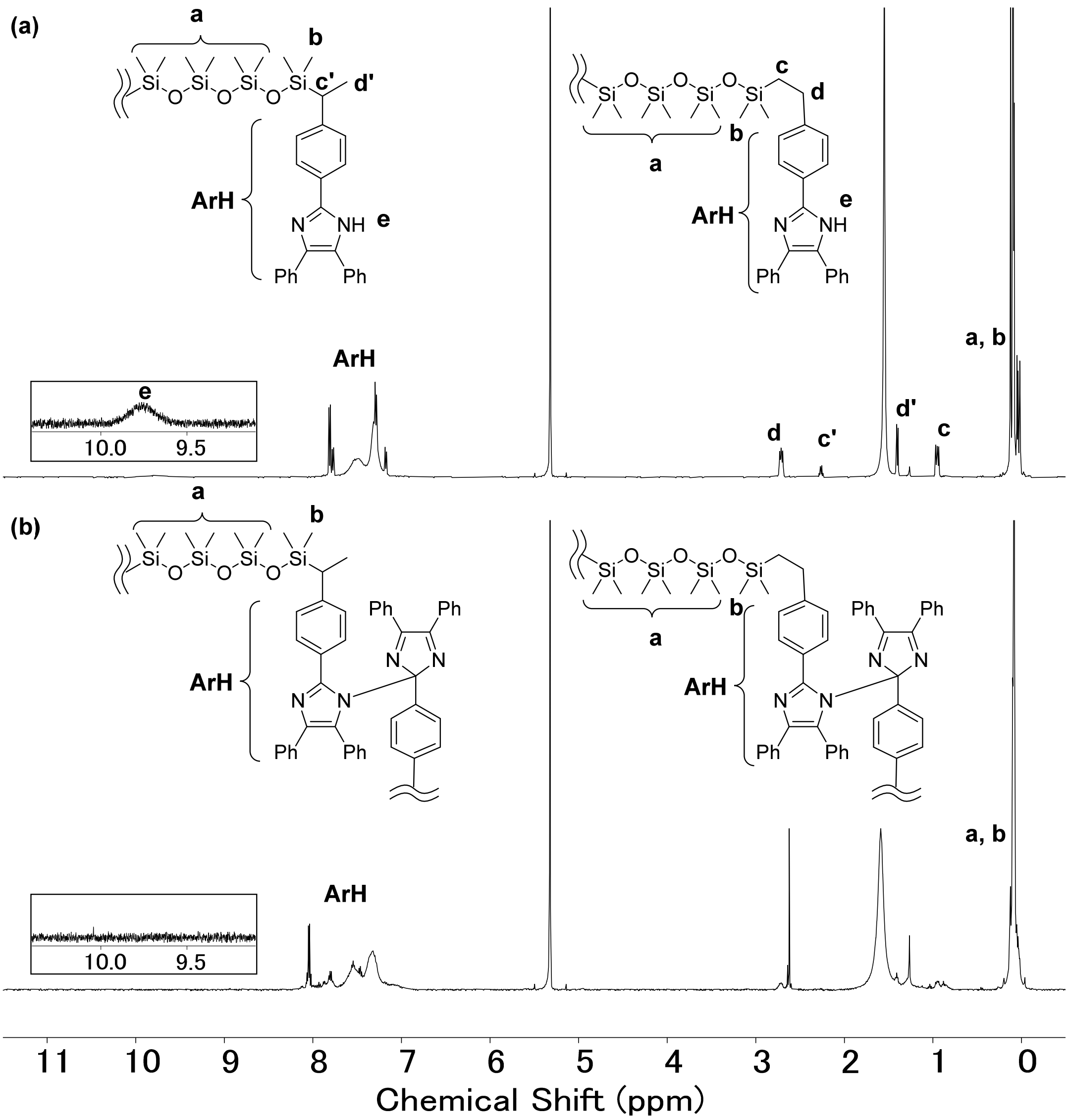

Supplementary Figure 5. Characterization of dimerization based on NMR spectrometry. 500 MHz ^{1}H NMR spectra of (a) $\mathbf{M_L}$ and (b) $\mathbf{M_C}$ (CD_2Cl_2).

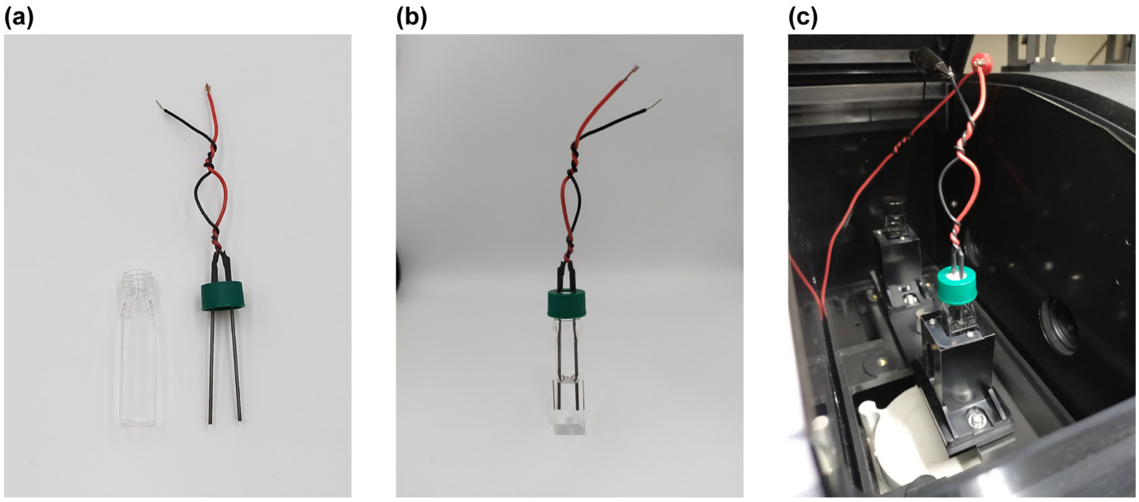

Supplementary Figure 6. *In situ* UV-vis measurement system for electrochemical oxidation. a, b Photographs of the separated quartz cell and screw cap equipped with mechanical pencil leads and the assembled measurement cell for the time-dependent analysis. **c** Photograph of the measurement cell set in UV-vis spectrometer and connected with lead wires.

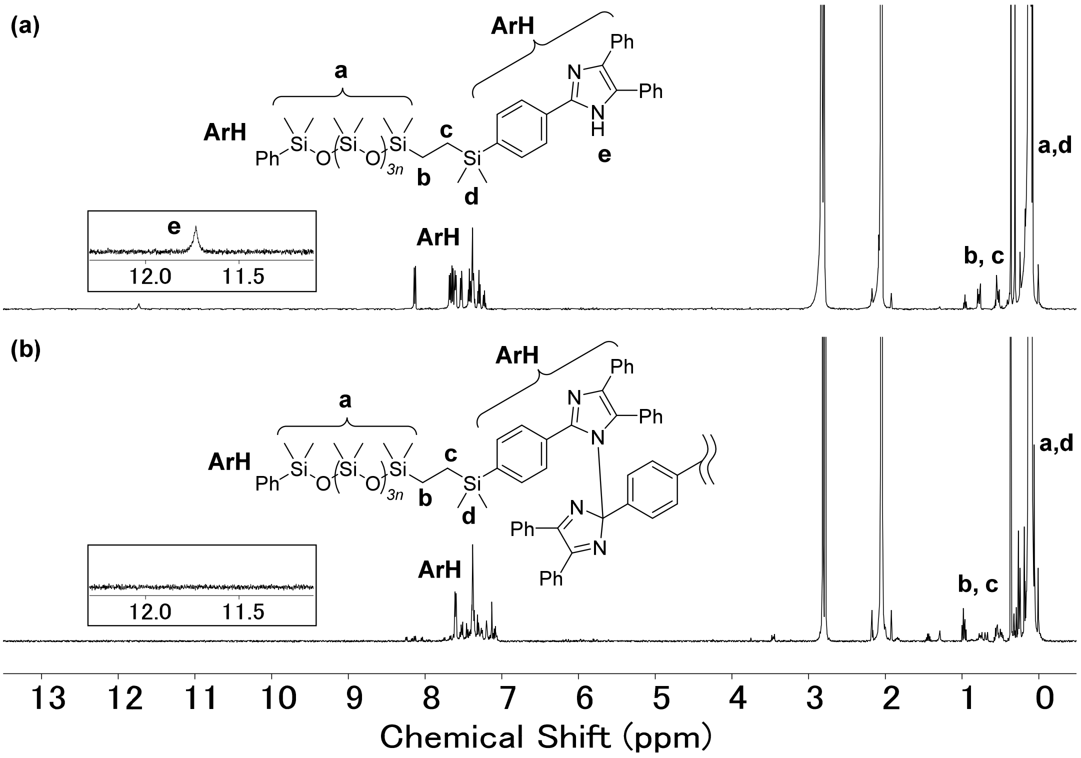

Supplementary Figure 7. Characterization of dimerization based on NMR spectrometry. 500 MHz ^{1}H NMR spectra of (a) $\mathbf{P_L}$ and (b) $\mathbf{P_D}$ (acetone-d_6).

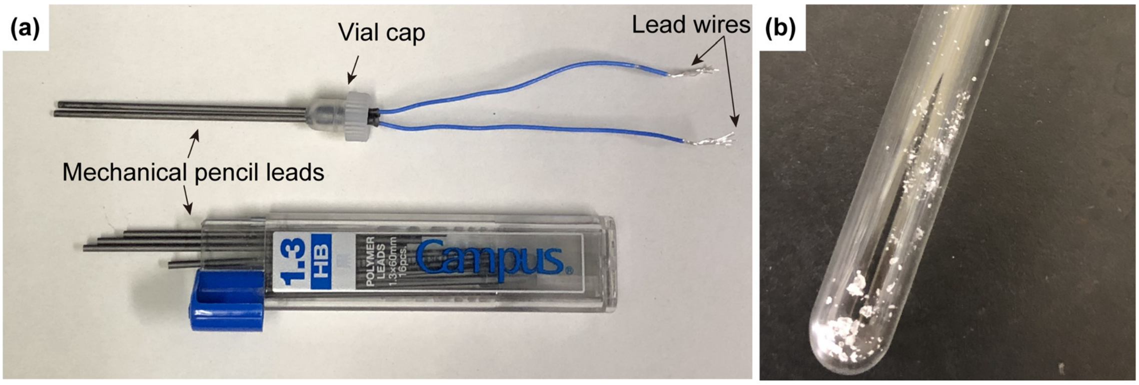

Supplementary Figure 8. Experimental setup up and specimen for electrochemical network formation. a Photograph of mechanical pencil leads equipped with a vial cap for superconvenient ETT system. **b** Phototograph of $\mathbf{S_L}$ used for the electrochemical network formation reaction.

Supplementary References

1. S. Honda, M. Oka, H. Takagi and T. Toyota, *Angew. Chem. Int. Ed.*, 2019, **58**, 144-148.
2. M. Oka and S. Honda, *React. Funct. Polym.*, 2021, **158**, 104800.
3. J. Beckmann, A. Duthie, G. Reeske and M. Schürmann, *Organometallics*, 2004, **23**, 4630-4635.
4. B. Lin and R. M. Waymouth, *J. Am. Chem. Soc.*, 2017, **139**, 1645-1652.
